# A cross-sectional observational study investigating the association between sedges (swamp grasses, Cyperaceae) and the prevalence of immature malaria vectors in aquatic habitats along the shore of Lake Victoria, western Kenya

**DOI:** 10.12688/f1000research.25673.2

**Published:** 2020-10-01

**Authors:** Getachew E. Bokore, Paul Ouma, Patrick O. Onyango, Tullu Bukhari, Ulrike Fillinger

**Affiliations:** 1 International Centre of Insect Physiology and Ecology (icipe), P.O. Box 30772–00100, Nairobi, Kenya; 2 School of Physical and Biological Sciences, Department of Zoology, Maseno University, P.O. Box 333 – 40105, Maseno, Kenya; 3 Public Health Entomology Team, Ethiopian Public Health Institute, P.O. Box 1242, Addis Ababa, Ethiopia

**Keywords:** Anopheles, oviposition, larval ecology, malaria, vector control, vegetation, graminoid plants

## Abstract

**Background**: Strategies that involve manipulations of the odour-orientation of gravid malaria vectors could lead to novel attract-and-kill interventions. Recent work has highlighted the potential involvement of graminoid plants in luring vectors to oviposition sites. This study aimed to analyse the association between water-indicating graminoid plants (Cyperaceae, sedges), other abiotic and biotic factors and the presence and abundance of early instar *Anopheles* larvae in aquatic habitats as a proxy indicator for oviposition.

**Methods**: A cross-sectional survey of 110 aquatic habitats along the shores of Lake Victoria was done during the rainy season. Habitats were sampled for mosquito larvae using the sweep-net method and habitat characteristics recorded.

**Results**: *Anopheles arabiensis* was the dominant species identified from aquatic habitats. Larvae of the secondary malaria vectors such as *Anopheles coustani, An. rufipes *and *An. maculipalpis *were found only in habitats covered with graminoids, whereas *An. arabiensis, An. ziemanni* and *An. pharoensis *were found in both habitats with and without graminoid plants. The hypothesis that sedges might be positively associated with the presence and abundance of early instar *Anopheles* larvae could not be confirmed. The dominant graminoid plants in the habitats were *Panicum repens*, *Cynodon dactylon* in the Poaceae family and *Cyperus rotundus *in the Cyperaceae family. All of these habitats supported abundant immature vector populations. The presence of early instar larvae was significantly and positively associated with swamp habitat types (OR=22, 95% CI=6-86, P<0.001) and abundance of late *Anopheles* larvae (OR=359, CI=33-3941, P<0.001), and negatively associated with the presence of tadpoles (OR=0.1, CI=0.0.01-0.5, P=0.008).

**Conclusions**: Early instar malaria vectors were abundant in habitats densely vegetated with graminoid plants in the study area but no specific preference could be detected for any species or family. In search for oviposition cues, it might be useful to screen for chemical volatiles released from all dominant plant species.

## Background

Malaria, despite increased control efforts, is still among the leading human diseases in Africa. In 2018, 213 million people were infected and 380,000 died ^1^. The majority of people in sub-Saharan Africa live in poverty and in areas with suitable conditions for the proliferation of malaria vectors ^2^. The major malaria vectors are in the *Anopheles gambiae* and *An. funestus* species complexes, but a number of less efficient, so-called secondary vectors also contribute to malaria transmission ^3, 4^.

With growing physiological and behavioural resistance of malaria vectors to insecticides ^5– 7^, research efforts are geared towards additional, non-insecticidal vector control strategies ^8, 9^. Manipulations of the odour-orientation of adult vectors could lead to novel attract-and-kill interventions ^10– 12^. The gravid female searching for a suitable oviposition site is a desirable target for control. This strategy is specifically important as a single gravid mosquito may lay between 50 to 150 eggs ^13^, hence killing a single gravid mosquito affects the growth of the population. Understanding the cues for habitat selection is of paramount importance for the development of such a tool. Recent work has highlighted the potential involvement of graminoid plants in luring vectors to oviposition sites ^14^. It has, for example, been shown that *Anopheles* mosquitoes respond to volatile chemical compounds that emanate from rice plants ^15^.

Malaria vector mosquitoes lay their eggs in standing water and grass-like (graminoid) plants that often dominate wetlands associated with high *Anopheles* larval densities ^16– 18^. Some graminoid plants, similar to lowland rice ( *Oryza sativa*), are well adapted to wetlands. Most species in the sedge family, also known as swamp grasses, (Cyperaceae) are wetland indicators. One sedge species, *Cyperus rotundus*, was recently associated with the discovery of the oviposition attractant cedrol ^19^ but its connection to the sesquiterpene compound was not clearly understood.

We considered it plausible to hypothesize that there might be an association between chemical cues released by water-indicating plants that are used by gravid malaria vectors in search of suitable oviposition sites. Therefore, we implemented this study driven by the hypothesis that sedges (Cyperaceae) are associated with the presence and abundance of early instar *Anopheles* larvae, as a proxy indicator for oviposition, in western Kenya.

Swamp habitats are very common along lakeshores and serve as permanent or semi-permanent breeding sites for malaria vectors ^20^. Studies support that the abundance of *Anopheles* larvae are associated with habitats surrounded by grass-like plants ^14^. In the current study, we investigated: (1) the distribution of graminoid plants associated with aquatic habitats along the shores of Lake Victoria in western Kenya, (2) the association of the graminoid plants with the occurrence and abundance of *Anopheles* larvae, and (3) the association of abiotic and biotic factors in aquatic habitats with the occurrence and abundance of *Anopheles* larvae.

## Methods

### Study area

**Figure 1.**
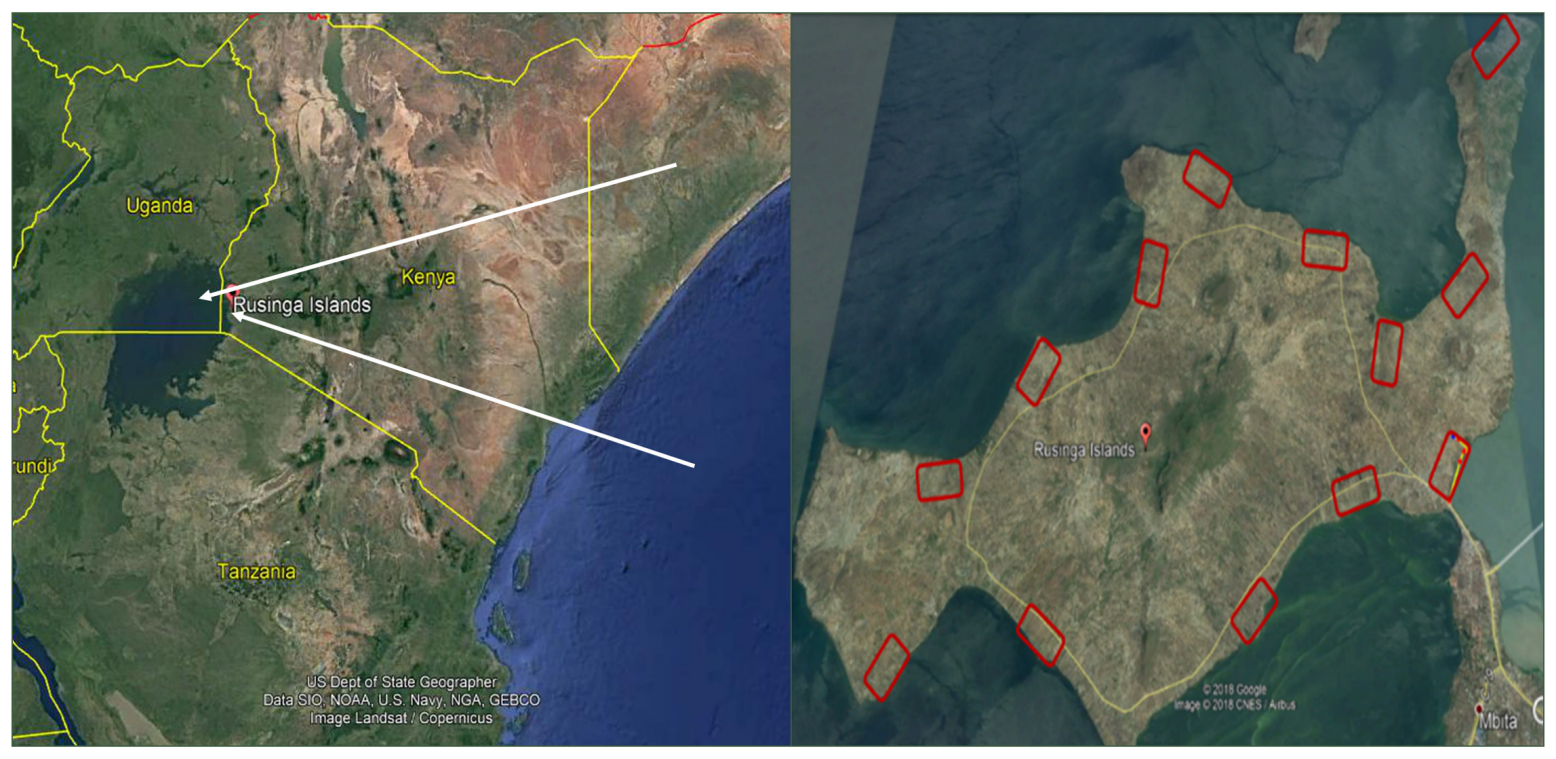
Map showing ( **A**) Lake Victoria region, East Africa ( **B**) the study clusters (rectangles in red) along the shores of Lake Victoria on Rusinga Island (Source: Google Earth).

This study was conducted on Rusinga Island (0°21′ and 0°26 south, 34°13′ and 34°07′ east) along the shore of Lake Victoria in Homa Bay County, western Kenya ^21^ ( Figure 1). The area is endemic for malaria and the estimated prevalence of malaria in the population of Rusinga Island is around 10% ^18, 22, 23^. Rusinga Island is only around 100 metres away from the mainland and connected via a bridge. The island has an area of 44 km ^2^ with altitude ranging from 1100 m to 1300 m above sea level and a population size of about 25,000 ^24^. The daily average temperatures of Rusinga Island range from 16°C to 34°C and peak in dry seasons ^24^. The area experiences bimodal rainy seasons with long rains from March to June and short rains from November to December. Malaria transmission peaks following the end of the long rainy season in June/July ^25^. The field survey was implemented between May and June 2018, towards the end of the long rainy season.

Habitat surveys were done along stretches of 700 m long and 300 m wide (clusters of approx. 0.2 km ^2^). A total of 13 sampling clusters were selected around the lake shores of Rusinga Island ( Figure 1B). The areas were selected with the help of Google Earth, aiming at a homogeneous distribution around the island. Inaccessible areas with steep rocks at the shoreline were excluded. Within each sampling cluster all aquatic habitats’ locations were recorded using a smartphone, a unique identifier allocated and sampled as outlined below.

### Aquatic habitat surveys

The aquatic habitat types were categorized as either swamp, puddle, fishpond, drainage/trench or artificial pit. The perimeter of every habitat was estimated, always by the same field worker for uniformity, by walking in large steps around the habitat. Water turbidity was measured using a turbidity meter (TRB 355IR, WTW Germany) and water pH and temperature were measured using a portable multi-parameter probe (Multi, WTW Germany). These parameters have been shown to be associated with larvae based on previous work done in the same study area..

Every aquatic habitat was inspected for the presence of larvae using the sweep-net method as described by Ndenga and others ^27^. The sweep-net (40 cm × 15 cm × 30 cm) was made from fine cotton cloth with a 150 cm long handle. It was chosen for sampling due to its better efficiency in sampling the diverse aquatic fauna including freshly hatched mosquito larvae and mosquito pupae than the standard dipper ^27, 28^. A dipper was used for sampling when the habitat was too small to be sampled by a sweep-net. Sampling of mosquito larvae using either sampling tools was randomly done at different points of the habitats since different species of malaria vectors prefer different conditions and vegetations. The duration of sweeping was dependent on the perimeter of the habitat. About 10 minutes were taken to sweep habitats with perimeters exceeding 10 metres, while 5 minutes were taken to sweep habitats <10 m in perimeter. All sweeps were emptied into white trays and mosquito immature stages were counted separately for the two encountered genera, *Anopheles* and *Culex*. *Culex* and *Anopheles* larvae were identified morphologically. *Culex* larvae possess a siphon on the posterior part of their abdomen for breathing whereas *Anopheles* larvae have no siphon and rest horizontal to the water body ^29^. The larvae were grouped as early (1 ^st^ and 2 ^nd^ instar) and late (3 ^rd^ and 4 ^th^ instar) instars based on their body size. In addition, macroinvertebrates sampled from a habitat were grouped as Odonata (dragonfly and damselfly larvae), Coleoptera (water beetle larvae and adults), Heteroptera (Notonectidae, Naucoridae and Nepidae), fish and tadpoles. All late instar *Anopheles* larvae and mosquito pupae were transferred to water bottles (1 L) containing habitat water and taken to the International Centre of Insect Physiology and Ecology-Thomas Odhiambo Campus ( *icipe*-TOC) for rearing to adults. Rearing of the field collected larvae was done in 1 L plastic containers. Larvae were fed with a pinch of ground dry cat food (Nestlé Purina PetCare Company, Nairobi, Kenya) once daily. The emerged adults were killed in a -20°C refrigerator, sorted by genera and all *Anopheles* adults stored in Eppendorf tubes (1.5 ml) at -71°C until they were identified morphologically using printed keys ^30^ and molecularly using polymerase chain reaction (PCR) followed by gel-electrophoresis ^31^. Randomly selected mosquito samples were used for molecular identification. Polymerase chain reaction was implemented for the amplification of the ribosomal internal transcribed spacer (ITS2) gene using primers ^32^. Positive controls of *An. gambiae s.s.* and *An. arabiensis* (from the insectary) were analyzed with the samples from the field. Extraction of DNA was done for each mosquito separately using Tissue Kit (Quagen, GmbH Hilden, Germany). The PCR was prepared by mixing PCR mix of 2 µl of 5XHot Firepol Blended Master Mix (Ready to Load), primers (0.5 µM each), DNA template (2 µl) and nuclease-free water (5 µl). The thermal recycling conditions involved initial denaturation for 5 min at 95°C, after which 30 cycles of denaturation followed for 30 s at 94°C, annealing for 30 s at 50°C, extension for 30 s at 72°C and final extension for 5 min at 72°C. We used a Kyratec Thermal Cycler (SC300T-R2, Australia) for the thermal reactions. Agarose gel-electrophoresis (2.0%) stained with 2 µl ethidium bromide against a 100 bp DNA ladder (Bioline, A Maridian Life Science ^@^ Company, UK) and a positive control was conducted to identify the species.

Vegetation coverage, vegetation types and the dominant vegetation type were recorded separately for habitat edge and water surface. Habitat edge was defined as the area along the waterline, approximately 10 cm inside and/or outside the water. Vegetation coverage was estimated visually, always by the same field worker, as the proportion of the habitats covered with vegetations and categorized as (1) 1–25% (2) 25–50% (3) 50–75% (4) 75–100%. Graminoid plants across the edge and inside water were recorded as Poaceae, Cyperaceae, Juncaceae, Typhaceae. The graminoid plants were identified to family using the morphology of their leaves (two or three-ranked; open or closed sheaths), and their stem type (three-sided or round; hollow or solid) using Revuelta ^33^. Furthermore, herbaceous (not woody and non-graminoid plants) were collectively recorded as forbs. The presence of water plants and algae in the aquatic habitats was also recorded. The percent coverage of water plants and algae on the water surface was visually determined as above. For each habitat, the dominant type of vegetation was identified and recorded. Full specimens of all dominant graminoid plants found in the aquatic habitats were collected and planted at *icipe-*TOC for further identification ^34, 35^.

### Data analysis

Generalised estimating equations (GEE) with Poisson distribution fitted to a log function and exchangeable correlation matrix were used to test for associations between biological and environmental factors and the abundance of early instar *Anopheles* larvae. The cluster ID in which habitats were located was included in the model as repeated measurement. A GEE model was also used to analyse associations between factors and the presence of early instar *Anopheles* larvae. Here we included the presence of early instar *Anopheles* larvae as dependent variable in the model with binomial distribution fitted to a logit function and exchangeable correlation matrix to analyse its association with biotic and abiotic factors of the habitats (independent variables). The presence and abundance of early instar *Anopheles* larvae (rather than eggs which are difficult to identify from field samples) were used as dependent variable as a proxy for oviposition. This is based on recent work confirming that early instar density correlates with the abundance of females selecting a habitat for oviposition ^36^. The statistical outputs were reported as incidence rate ratios (RR) for the abundance of the first instar larvae and odds ratios (OR) for the presence with their 95% confidence intervals (95% CI). R statistical software version 3.5.1 ^37^ was used for the analyses.

### Ethics statement

This field survey was largely descriptive and observational and had no human study participants. Habitat surveys on privately owned lands were made after seeking consent from the landowners and were implemented in their presence.

## Results

### Aquatic habitat types

**Figure 2.**
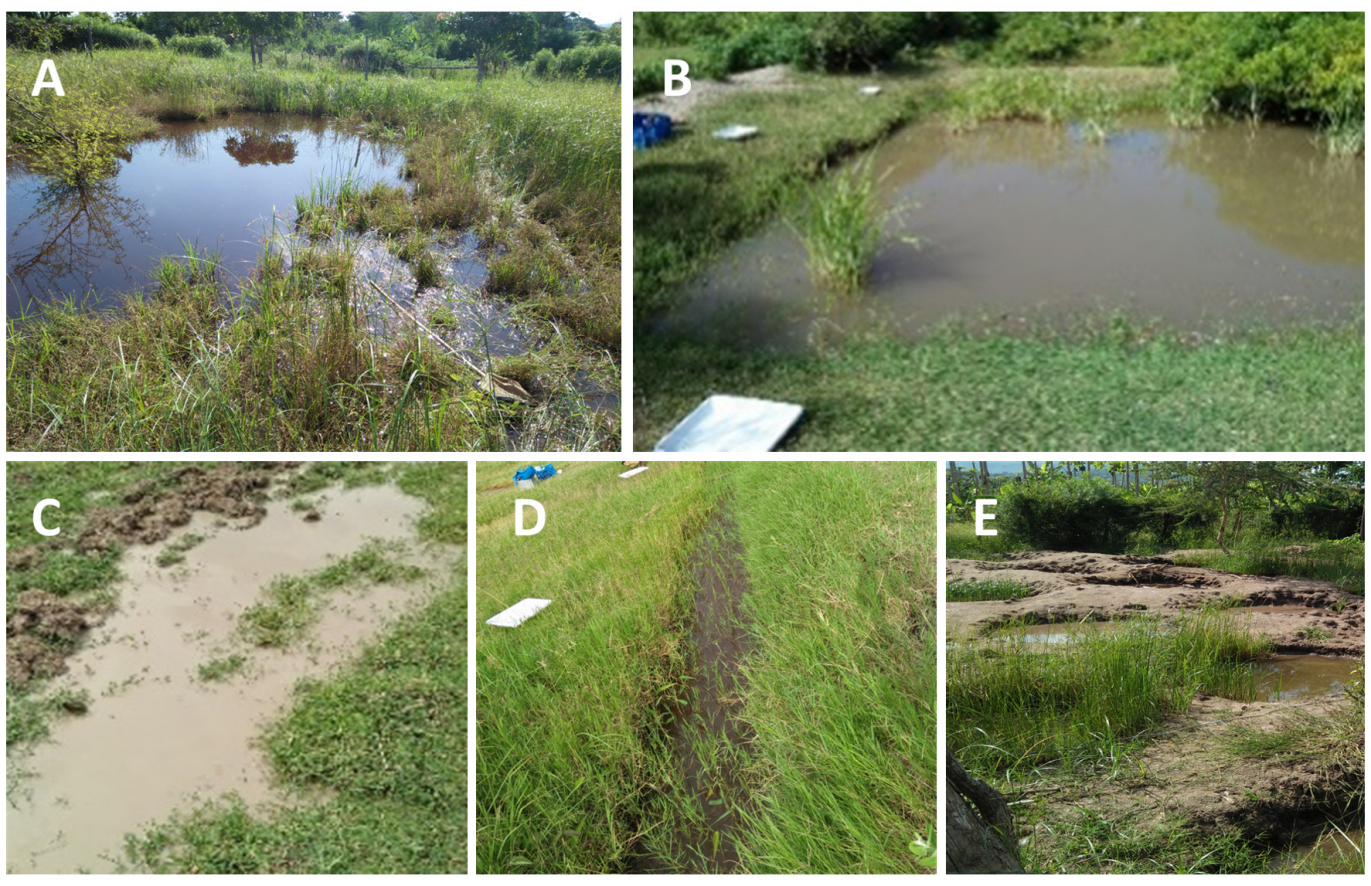
Examples of habitat types. ( **A**) Swamp, ( **B**) Fishpond, ( **C**) Puddle, ( **D**) Drainage, ( **E**) Artificial pit.

**Figure 3.**
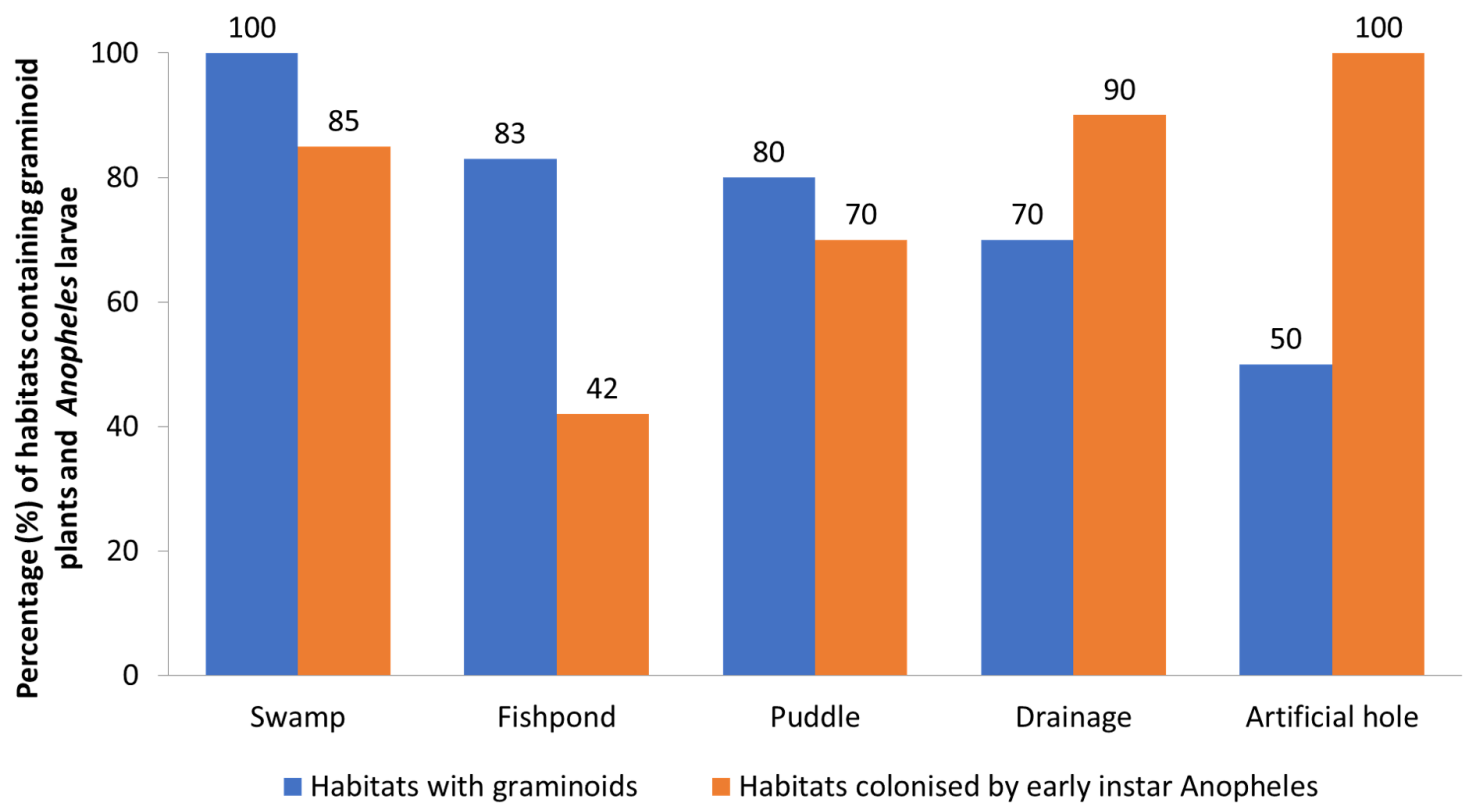
Bar graph showing percentage of habitats containing graminoid plants and being colonised by early instar *Anopheles* larvae.

A total of 110 aquatic habitats were identified during the survey ^38^. As expected, given the targeted areas within 300 metres of the lake shore, the most prevalent aquatic habitat types were swamps (65.5%, n=72) defined as permanent or semi-permanent water-logged sections of land with tall graminoid vegetation and/or floating plants ( Figure 2A). The water sources of these were largely groundwater supplemented by rainwater. Other habitats (see Figure 2B, Figure 3C, 3D and 3E) included ponds formerly used for breeding fish but abandoned at the survey time (11%, n=12), rainfed puddles (9%, n=10), drainages (9%, n=10) and artificial pits (5.5%, n=6). Given that all non-swamp habitats were few in number, they were pooled for statistical analysis and the swamp habitats used as the reference group ( Figure 3). Early instar *Anopheles* larvae were found frequently during the survey in the habitat types: artificial pits (n=6, 100%), drainages (n=9, 90%), ponds (n=5, 42%), puddles (n=7, 70%) and swamps (n=61, 85%). The majority of these habitat types were characterized by possessing graminoid plants: graminoid plants dominated the vegetation in 100% of the swamps, in 83% of the ponds, in 80% of the puddles, in 70% of the drainages, and in 50% of the artificial pits.

### Association between graminoid plants and the presence and abundance of *Anopheles* larvae

**Figure 4.**
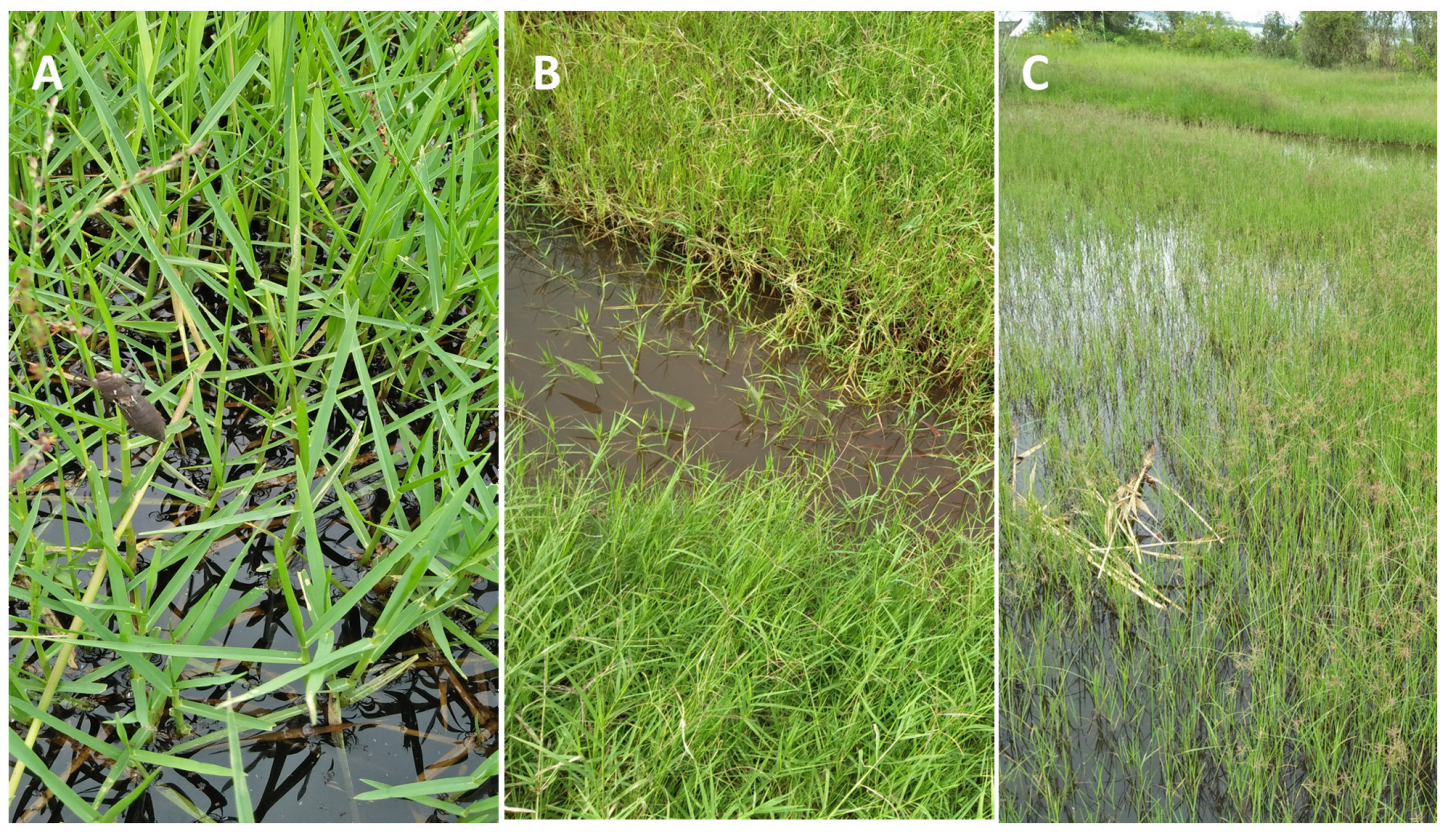
The most dominant graminoid plants identified during the survey. ( **A**) *Panicum repens* (Poaceae), ( **B**) *Cynodon dactylon* (Poaceae) and ( *C*) *Cyperus rotundus* (Cyperaceae).

All the swamp habitats were bordered by graminoid plants along the water edges and had a high surface coverage. Similarly, 84% (32/38) of non-swamp habitats had graminoids along their edges and 76% (29/38) had graminoids at their surfaces. Unexpectedly, swamp grasses were not the most frequently found graminoid plants in the survey. Representatives of the Cyperaceae family were found only in 39% of the aquatic habitats sampled. Among the Poaceae family, torpedo grass ( *Panicum repens)* and Bermuda grass ( *Cynodon dactylon*) were the dominant species ( Figure 4).

Of the surveyed habitats, 42 (38%) were found covered by *P. repens* along their edges and 47 (43%) of the habitats at their surfaces. *Cynodon dactylon* was found covering the habitats both along the edges in 35 (32%) habitats and surfaces in 25 (23%) habitats ( Table 1). Overall, graminoid plants dominated in 96 habitats whilst forbs dominated only in five habitats during the survey. Nine habitats had no vegetations at their surface and five of them were colonized by early instar *Anopheles* larvae. We found water plants in 26 (24%) out of the 110 habitats and most (n=20, 77%) of them in swamp habitats. Filamentous algae were recorded in 21 habitats. Contrary to our hypothesis, there was no significant association between the presence or abundance of early instar *Anopheles* larvae and the dominant graminoid plant present in a habitat ( Table 1).

**Table 1.** Association between dominant graminoid plants, and the presence and abundance of *Anopheles* early instar larvae.

Factor	No. habitats	Mean (95% CI) of *Anopheles* early instar larvae	Presence of *Anopheles* early instar larvae		Abundance of *Anopheles* early instar larvae	
			OR (95% CI)	P value	RR (95% CI)	P value
*Cyperus rotundus* (Cyperacea) *	14	57 (22.19-149)	1		1	
*Cynodon dactylon* (Poaceae)	25	99 (48-205)	1.1 (0.7-1.7)	0.762	1.7 (0.6-5.5)	0.35
*Panicum repens* (Poaceae)	47	84 (48-146)	1.2 (0.9-1.7)	0.305	1.5 (0.5-4.2)	0.99
Others (Poaceae)	10	58 (33-101)	1.4 (0.99-2)	0.057	1.01 (0.3-3)	0.48

*Selected as reference based on initial hypothesis and earlier association of *Cyperus rotundus* with oviposition. OR= odds ratio, RR= rate ratio, CI= confidence interval.

### *Anopheles* species composition

A total of 14,145 early and late instar *Anopheles* larvae and 402 pupae were collected. Out of those, 4,650 emerged into adults and were morphologically identified ( Table 2). *Anopheles gambaie s.l.* represented 96% of all *Anopheles* specimen collected. Molecular identification was done for a random sample of 10% of the *An. gambiae s.l*. (n=480) and revealed 100% *An. arabiensis*.

*Anopheles coustani, An. rufipes and An. maculipalpis* were found only in aquatic habitats covered with graminoid plants, whereas *An. arabiensis, An. ziemanni* and *An. pharoensis* were found in both habitats with and without graminoid plants. All six species of *Anopheles* mosquitoes were recorded in swamp habitats. All of these *Anopheles* species were found in aquatic habitats with dense graminoid vegetation (50–100%) ( Table 3). However, only three species of *Anopheles* mosquitoes ( *An. arabiensis*, *An. ziemanni,* and *An. pharoensis*) were collected in habitats sparsely (1–25%) covered by graminoid plants.

**Table 2.** Species composition of *Anopheles* collected from habitats along the lake shore of Rusinga Island.

*Anopheles spp*	Number of mosquitoes	Percent composition
An. Arabiensis *	4481	96.24
*An. coustani*	22	0.47
*An. maculipalpis*	2	0.04
*An. pharoensis*	67	1.44
*An. rufipes*	27	0.58
*An. ziemanni*	57	1.22

* Molecular identification of a random sample of 10% of the *An. gambiae s.l*. revealed 100% *An. arabiensis*.

**Table 3.** Mean number ± 95% CI of different mosquito species in swamp and non-swamp habitats, habitats with and without Cyperaceae and graminoids coverage levels.

Factor	Variable	Mean (95% CI) of *Anopheles* mosquitoes identified from adults emerged from collected immature stages					
		*An.* *gambiae*	*An.* *ziemanni*	*An.* *coustani*	*An. pharoensis*	*An.* *maculipalpis*	*An. rufipes*
Habitat type	Non-swamp	30 (17-46)	0.3 (0.1-0.9)	0.3 (0.1-0.8)	0.4 (0.2-0.9)	0	0.5 (0.1-5)
	Swamp	45 (29-69)	0.6 (0.3-1.4)	0.1 (0.1-0.3)	0.7 (0.4-1.4)	0.03 (0-0.1)	0.1 (0-0.6)
Graminoids	No- Cyperaceae	41 (26-65)	0.5 (0.2-1.1)	0.2 (0.1-0.5)	0.4 (0.2-0.9)	0.03 (0-0.1)	0.4 (0.1-2)
	Cyperaceae	38 (22-66)	0.6 (0.2-1.8)	0.1 (0.1-0.4)	0.9 (0.4-1.9)	0	0.02 (0-0.4)
Graminoids coverage (%)	1-25%	48 (20-116)	0.1 (0.1-0.7)	0	0.13 (0.1-0.7)	0	0
	25-50%	69 (22-212)	0.2 (0-2)	0.7 (0.2-3)	0.7 (0.14-3.4)	0	0.10 (0-10)
	50-75%	46 (19-112)	0.1 (0.0-0.7)	0.1 (0-0.7)	0.2 (0.04-0.9)	0.1 (0-0.5)	0.3 (0-8)
	75-100%	31 (20-50)	0.9 (0.4-1.9)	0.2 (0.1-0.5)	0.9 (0.5-1.7)	0.02 (0-0.1)	0.4 (0.1-2)

CI= confidence interval.

Aquatic habitats populated with *Panicum repens* and forbs at their edges had all the six *Anopheles* species identified ( Table 4a). *Megaloprotachne albescens* was found dominant in six out of 110 habitats surveyed but was found to have all the six different species of *Anopheles* ( Table 4b). *Anopheles arabiensis*, *An. coustani,* and *An. pharoensis* were coexisting with all the graminoid types and forbs found dominating along the surfaces of the habitats.

**Table 4.** Dominant vegetations (a) at the edges and (b) at the surfaces and mean number of *Anopheles* mosquitoes. (a)

Dominant vegetation at habitat edge	No. of habitats	Mean (95% CI)					
		*An.* *arabiensis*	*An.* *ziemanni*	*An.* *coustani*	*An.* *pharoensis*	*An.* *maculipalpis*	*An. rufipes*
*Cyperus rotundus*	12	38 (14-108)	0.25 (0.03-2)	0.2 (0.03-1)	0.42 (0.1-2)	0	0
*Cynodon dactylon*	35	32 (18-59)	0.4 (0.1-1)	0.3 (0.1-1)	0.8 (0.3-2)	0	0.1 (0.01-1)
*Panicum repens*	42	44 (25-76)	0.9 (0.4-2)	0.1 (0-0.4)	0.8 (0.4-2)	0.02 (0-0.2)	0.02 (0-0.3)
Forbs	11	26 (9-77)	0.1 (0.01-1)	0.2 (0.03-1)	0.1 (0.01-1)	0.1 (0.01-0.7)	0.4 (0.02-6)
Others	7	76 (20-293)	0.1 (0.01-2)	0.1 (0.01-2	0.3 (0.03-2)	0	3 (0.01-66)

**Table:** (b)

Dominant vegetation covering habitat surface	No. of habitats	Mean (95% CI)					
		*An.* arabiensis*	*An.* *ziemanni*	*An.* *coustani*	*An.* *pharoensis*	*An.* *maculipalpis*	*An.* *rufipes*
*Cyperus rotundus*	14	45 (18-117)	0.1 (0.02-1)	0.1 (0.03-1)	1 (0.2-4)	0	0
*Cynodon dactylon*	25	25 (13-52)	0.2 (0.04-1)	0.1 (0-0.5)	0.4 (0.1-1.4)	0	0
*Panicum repens*	47	40 (24-67)	1 (0.4-2)	0.2 (0.1-0.4)	1 (0.3-2)	0.02 (0-0.2)	0.1 (0-0.3)
Forbs	5	28 (6-137)	0	0.4 (0.1-4)	0.2 (0.01-4)	0	0
Others	10	74 (24-227)	0.5 (0.1-4)	1 (0.2-3)	0.6 (0.1-3)	0.01 (0-0.2)	2 (0.3-0.3)

CI= confidence interval.

### Association between aquatic habitat biotic and abiotic factors and *Anopheles* larvae presence and abundance

The presence of early instar *Anopheles* larvae in habitats was significantly and positively associated with swamp-type habitats (OR=22, 95%CI=6-86, P<0.001), presence of late instar *Anopheles* larvae (OR=359, CI=33-3941, P<0.001), and presence of *Culex* larvae (OR=17, 95%CI=3-107, P=0.002) ( Table 5). In habitats containing pupae the odds of finding early instar *Anopheles* larvae was lower (OR=0.08, CI=0.01-0.42, P=0.003) than habitats without pupae. Notably, the majority of habitats with *Anopheles* larvae were also well colonised by other invertebrates, many of which are considered predators of mosquitoes, such as Odonatan, Notonecta, and Coleoptera larvae. However, the presence of early instar *Anopheles* was only significantly and negatively associated with presence of tadpoles (OR=0.09, 0.01-0.53, P=0.003). Correspondingly, the abundance of early instar *Anopheles* larvae significantly decreased with the presence of tadpoles (RR=0.5, CI=0.2-0.9, P=0.03). Similarly, larval abundance was negatively associated with presence of Odonata (RR=0.5, CI=0.3-0.9, P=0.019) and presence of Coleoptera (RR=0.4, CI=0.2-0.8, P=0.004). There was no significant association between the presence and abundance of early instar *Anopheles* larvae and habitat size, habitat depth, distance to the nearest house, water pH, water turbidity, biofilm, debris, algae, and water plants.

**Table 5.** Output of multivariate analysis of the presence or abundance of early instar *Anopheles* larvae as outcome, and biotic and abiotic factors as explanatory variables.

Factor	Category	Number of habitats	Larval presence/ absence		Larval abundance	
			OR (95 % CI)	P value	RR (95 % CI)	P value
**Abiotic factors**						
Habitat type	Non-swamp	38	1		1	
	Swamp	72	22 (6-86)	**<0.001**	1 (0.6-2)	0.625
Perimeter (m)	<50	84	1		1	
	≥50	26	0.3 (0.04-2.3)	0.249	0.9 (0.4-2)	0.754
Turbidity (NTU)	<200	90	1		1	
	≥200	20	1 (0.1-17)	0.780	2 (0.9-4)	0.099
**Biotic factors**						
*Anopheles* late instar		82	359 (33-3941)	**<0.001**	0.9 (0.4-2)	0.839
*Culex* larvae		88	17 (3-107)	**0.002**	2 (0.6-6)	0.244
Pupae		34	0.08 (0.01-0.42)	**0.003**	1 (0.7-2)	0.437
Odonata		41	2 (0.3-11)	0.518	0.5 (0.3-0.9)	**0.019**
Coleoptera		95	0.3 (0.03-3)	0.274	0.4 (0.2-0.8)	**0.004**
Fishes		38	0.4 (0.05-2)	0.288	0.6 (0.2-2)	0.336
Tadpoles		33	0.1 (0.01-0.5)	**0.008**	0.5 (0.2-0.9)	**0.030**

OR= odds ratio, RR= rate ratio, CI= confidence interval.

## Discussion

The work presented here was done with the aim of identifying graminoid plants for further behavioural and chemical ecology studies due to their association with habitats used by gravid malaria vectors for egg-laying. However, the presence of early instar *Anopheles* larvae in the majority of the surveyed habitats and the presence and high coverage of various graminoid plants did not allow us to analyse any statistically significant association. All the habitats surveyed provided excellent oviposition sites and favourable conditions for the development of immature stages based on the high and consistent number of early instar larvae as a proxy for oviposition and the associated high abundance of late instar larvae as an indicator for survival. The study, as implemented, did not allow us to infer specific plant-based factors with oviposition. Generally, the association between graminoid plants and *Anopheles* breeding sites as well as the presence and increased densities of *Anopheles* larvae in both temporary and permanent aquatic habitats have been shown before ^16, 17^. Recent studies have also shown promising odour-blends of volatile organic compounds identified from domesticated grasses such as rice and pollens of maize and sugarcane. These compounds, which include limonene, α-pinene, *p*-cymene, nonanal, benzaldehyde, sulcatone, β-caryophyllene, decanal, and 3-carene, have been reported in varieties of blend forms to elicit a response in gravid *An. arabiensis* ^15, 39, 40^. It has been suggested that vegetation can protect mosquito immature stages from being washed off by running water ^41^ and from predation ^42, 43^.

Our study has several limitations that might be responsible for the negative results. The timing of the survey towards the end of the rainy season meant that all potential habitats were flooded and vegetation thrived. Habitats for oviposition were not a limiting factor and likely easy to identify without major cues for orientation. This might have been different if the survey had been implemented during the dry season. Furthermore, this survey was limited to locations close to the lake shores, biasing the study towards swampy habitats. Potentially a more rigorous evaluation of the plant coverage using standard methods such as a quadrant frame which might have provided more detailed information on plant numbers, could have revealed more associations. However, given the high colonisation during the rainy season such method would be better applied during drier seasons. Lastly, due to high water levels during the peak rainy season, a number of habitats with swamp graminoids of the families Cyperaceae, Typhaceae, and Juncaceae were impossible to access, hence could not be sampled. This might also explain why only very few secondary malaria vector species and no *Anopheles funestus* were sampled, even though *An. funestus* is the major vector in houses in the study area ^44– 46^.

 Not many strong associations were found with early *Anopheles* larvae presence or abundance and other observed factors that would allow conclusions on oviposition preferences. However, the odds of finding early instars increased when late instar *Anopheles* larvae were present as opposed to when they were absent, potentially indicating that *Anopheles arabiensis* females oviposit in habitats containing late instar conspecific larvae as an indicator of suitable development conditions. This contrasts with experimental studies on *An. coluzzii* ^47, 48^, where it has been suggested that late instar conspecific larvae repel gravid females potentially due to the risk of cannibalism ^49^.

The presence and abundance of early instar *Anopheles* larvae were negatively associated with the presence of tadpoles. It was previously shown that rainwater conditioned with tadpoles repelled gravid *An. gambiae* from oviposition in the laboratory ^50^. Mature tadpoles can prey on larvae ^51^ and might compete for resources in aquatic habitats ^52^. Our field survey indicated that early instar larvae were cohabiting with predatory invertebrates in most habitats. Whilst *Anopheles* larvae might be reduced by these organisms, as suggested by the negative association between *Anopheles* density and the presence of Odonata and Coleoptera, gravid females nevertheless did not avoid these habitats for oviposition. This finding agrees with studies elsewhere that have shown strong associations between the presence of anopheline larvae and high invertebrate diversity ^53^.

Six species of *Anopheles* mosquitoes were identified from the samples which have all been reported in previous studies in western Kenya ^18, 54, 55^. *Anopheles arabiensis* was the predominant malaria vector from both vegetated and non-vegetated aquatic habitats in the study area during the peak rainy season. *Anopheles arabiensis* has historically been the predominant vector species on Rusinga Island ^56^. In recent years however, *An. funestus* predominates indoor vector collections ^44, 46, 57^, but larvae were not found during our survey. Breeding sites preferred by this mosquito species were inaccessible by the field team due to the large volumes of water in the lake after the long rains; *An. funestus* prefers breeding habitats that are covered by tall vegetations ^38, 58, 59^.

## Conclusions

Our results did not support the hypothesis and nullified the aim of the research to identify graminoid plant species positively associated with malaria vector oviposition. Our results did not support our initial hypothesis and did not allow us to identify any association between *Anopheles* larvae and specific graminoid plants. However, *Panicum repens*, *Cynodon dactylon,* and *Cyperus rotundus* were the predominant graminoid plants found in the aquatic habitats. The habitats covered by these vegetations were abundantly colonized by early instar *Anopheles* larvae even though no specific preference for any of these could be detected, likely due to study limitations. We recommend further studies on the identification of oviposition cues from graminoid plants during the dry seasons when habitats are limited and water-levels low enough to provide access to most of them. Furthermore, it might be warranted to implement bioassays in the laboratory with the here identified grass-like plants, which will allow more standardised comparisons and sufficient replication.

## Data availability

### Underlying data

Harvard Dataverse: Association between graminoids and the prevalence of immature malaria. https://doi.org/10.7910/DVN/NAT0YY ^60^.

This project contains the following underlying data:

- Bokore *et al.* 2020_F1000Research_All_Collected_Data.csv
- Bokore *et al.* 2020_F1000Research_Data_used_for_final_analysis.csv
- Bokore *et al.* 2020_F1000Research_Variable_Codes.csv

Data are available under the terms of the Creative Commons Zero "No rights reserved" data waiver (CC0 1.0 Public domain dedication).

## References

[1] WHO: World Malaria Report 2019. Geneva: World Health Organization. 2019;1–232. Reference Source

[2] Muema JM Bargul JL Njeru SN: Prospects for malaria control through manipulation of mosquito larval habitats and olfactory-mediated behavioural responses using plant-derived compounds. *Parasit Vectors.* 2017;10(1):184. 10.1186/s13071-017-2122-8 28412962 PMC5392979

[3] Sinka ME Bangs MJ Manguin S: A global map of dominant malaria vectors. *Parasit Vectors.* 2012;5:69. 10.1186/1756-3305-5-69 22475528 PMC3349467

[4] Coetzee M Hunt RH Wilkerson R: *Anopheles coluzzii* and *Anopheles amharicus,* new members of the *Anopheles gambiae* complex.2013;3619(3):246–74. Reference Source 26131476

[5] Ranson H Lissenden N: Insecticide Resistance in African *Anopheles* Mosquitoes: A Worsening Situation that Needs Urgent Action to Maintain Malaria Control. *Trends Parasitol.* 2016;32(3):187–96. 10.1016/j.pt.2015.11.010 26826784

[6] Coleman M Hemingway J Gleave KA: Developing global maps of insecticide resistance risk to improve vector control. *Malar J.* 2017;16(1):86. 10.1186/s12936-017-1733-z 28222727 PMC5320685

[7] Cook J Tomlinson S Kleinschmidt I: Implications of insecticide resistance for malaria vector control with long-lasting insecticidal nets: Trends in pyrethroid resistance during a WHO-coordinated multi-country prospective study. *Parasit Vectors.* 2018;11(1):550. 10.1186/s13071-018-3101-4 30348209 PMC6198431

[8] Russell TL Beebe NW Cooper RD: Successful malaria elimination strategies require interventions that target changing vector behaviours. *Malar J.* 2013;12(1):56. 10.1186/1475-2875-12-56 23388506 PMC3570334

[9] Takken W Knols BG: Malaria vector control: current and future strategies. *Trends Parasitol.* 2009;25(3):101–4. 10.1016/j.pt.2008.12.002 19168392

[10] Lindh JM Okal MN Herrera-Varela M: Discovery of an oviposition attractant for gravid malaria vectors of the *Anopheles gambiae* species complex. *Malar J.* 2015;14(1):119. 10.1186/s12936-015-0636-0 25885703 PMC4404675

[11] Eneh LK Fillinger U Borg Karlson AK: *Anopheles arabiensis* oviposition site selection in response to habitat persistence and associated physicochemical parameters, bacteria and volatile profiles. *Med Vet Entomol.* 2019;33(1):56–67. 10.1111/mve.12336 30168151 PMC6359949

[12] Mbare O Lindsay SW Fillinger U: Testing a pyriproxyfen auto-dissemination station attractive to gravid *Anopheles gambiae sensu stricto* for the development of a novel attract-release -and-kill strategy for malaria vector control. *BMC Infect Dis.* 2019;19(1):800. 10.1186/s12879-019-4438-9 31510931 PMC6740013

[13] Lyimo Edith O Takken W: Effects of adult body size on fecundity and the pre-gravid rate of *Anopheles gambiae* females in Tanzania. *Med Vet Entomol.* 1993;7(4):328–32. 10.1111/j.1365-2915.1993.tb00700.x 8268486

[14] Asmare Y Hill SR Hopkins RJ: The role of grass volatiles on oviposition site selection by *Anopheles arabiensis* and *Anopheles coluzzii*. *Malar J.* 2017;16(1):65. 10.1186/s12936-017-1717-z 28173804 PMC5297170

[15] Wondwosen B Birgersson G Seyoum E: Rice volatiles lure gravid malaria mosquitoes, *Anopheles arabiensis*. *Sci Rep.* 2016;6(1):37930. 10.1038/srep37930 27901056 PMC5128813

[16] Fillinger U Sonye G Killeen GF: The practical importance of permanent and semipermanent habitats for controlling aquatic stages of *Anopheles gambiae sensu lato* mosquitoes : operational observations from a rural town in western Kenya. *Trop Med Int Health.* 2004;9(12):1274–89. 10.1111/j.1365-3156.2004.01335.x 15598259

[17] Imbahale SS Paaijmans KP Mukabana WR: A longitudinal study on *Anopheles* mosquito larval abundance in distinct geographical and environmental settings in western Kenya. *Malar J.* 2011;10(1):81. 10.1186/1475-2875-10-81 21477340 PMC3080801

[18] Minakawa N Dida GO Sonye GO: Malaria Vectors in Lake Victoria and Adjacent Habitats in Western Kenya. *PLoS One.* 2012;7(3):e32725. 10.1371/journal.pone.0032725 22412913 PMC3297610

[19] Eneh LK Saijo H Borg-Karlson AK: Cedrol, a malaria mosquito oviposition attractant is produced by fungi isolated from rhizomes of the grass *Cyperus rotundus.* *Malar J.* 2016;15(1):478. 10.1186/s12936-016-1536-7 27639972 PMC5027114

[20] Minakawa N Sonye G Dida GO: Recent reduction in the water level of Lake Victoria has created more habitats for *Anopheles funestus*. *Malar J.* 2008;7(1):119. 10.1186/1475-2875-7-119 18598355 PMC2490699

[21] Google Earth: Satellite view of Lake Victoria region: Study sites view of Rusinga Islands along the shore of Lake Victoria. Reference Source

[22] Noor AM Gething PW Alegana VA: The risks of malaria infection in Kenya in 2009. *BMC Infect Dis.* 2009;9(1):180. 10.1186/1471-2334-9-180 19930552 PMC2783030

[23] Olanga EA Okombo L Irungu LW: Parasites and vectors of malaria on Rusinga Island, Western Kenya. *Parasit Vectors.* 2015;8(1):250. 10.1186/s13071-015-0860-z 25928743 PMC4422414

[24] Homan T di Pasquale A Onoka K: Profile: The Rusinga Health and Demographic Surveillance System, Western Kenya. *Int J Epidemiol.* 2016;45(3):718–27. 10.1093/ije/dyw072 27185811

[25] Kagaya W Gitaka J Chan CW: Malaria resurgence after significant reduction by mass drug administration on Ngodhe Island, Kenya. *Sci Rep.* 2019;9(1):19060. 10.1038/s41598-019-55437-8 31836757 PMC6910941

[26] Harrera-Varela M: Larval habitat discrimination by the African malaria vector Anopheles gambiae sensu lato: observations from standardized experiments and field studies.Phd thesis.2015 10.17037/PUBS.02222109

[27] Ndenga BA Simbauni JA Mbugi JP: Productivity of Malaria Vectors from Different Habitat Types in the Western Kenya Highlands.Amdam GV editor. *PLoS One.* 2011;6(4):e19473. 10.1371/journal.pone.0019473 21559301 PMC3085476

[28] de Klerk AR Wepener V: The influence of biotope and sampling method on the assessment of the invertebrate community structure in endorheic reed pans in South Africa. *African J Aquat Sci.* 2011;36(1):67–74. 10.2989/16085914.2011.559705

[29] Snell AE: Identification keys to larval and adult female mosquitoes (Diptera: Culicidae) of New Zealand. *New Zeal J Zool.* 2005;32(2):99–110. 10.1080/03014223.2005.9518401

[30] Gillies MT Coetzee M: A Supplement to the Anophelinae of Africa South of the Sahara. *Publ South African Inst Med Res.* 1987;55(55):63 Reference Source

[31] Scott JA Brogdon WG Collins FH: Identification of single specimens of the *Anopheles gambiae* complex by the polymerase chain reaction. *Am J Trop Med Hyg.* 1993;49(4):520–9. 10.4269/ajtmh.1993.49.520 8214283

[32] Zianni MR Nikbakhtzadeh MR Jackson BT: Rapid discrimination between *Anopheles gambiae s.s.* and *Anopheles arabiensis* by High-Resolution Melt (HRM) analysis. *J Biomol Tech.* 2013;24(1):1–7. 10.7171/jbt.13-2401-001 23543777 PMC3518878

[33] Revuelta EA: Introduction to Florida’s Graminoids.2019;1–44. Reference Source

[34] Fish L Mashau AC Moeaha MJ: Identification guide to southern African grasses. An identification manual with keys, descriptions and distributions. In: *Pretoria: South African National Biodiversity Institute*;2015;1–30. Reference Source

[35] Muyekho FN Barrion AT Khan ZR: A Primer on grass identification and their uses in Kenya.2004;86 Reference Source

[36] Odero JO Fillinger U Rippon EJ: Using sibship reconstructions to understand the relationship between larval habitat productivity and oviposition behaviour in Kenyan *Anopheles arabiensis*. *Malar J.* 2019;18(1):286. 10.1186/s12936-019-2917-5 31443645 PMC6708163

[37] R Core Team: R: A Language and Environment for Statistical Computing. Vienna, Austria;2018 Reference Source

[38] Dia I Guelbeogo MW Ayala D: Advances and perspectives in the study of the malaria mosquito *Anopheles funestus*.In: *Anopheles mosquitoes - New insights into malaria vectors. *InTech;2013 10.5772/55389

[39] Wondwosen B Birgersson G Tekie H: Sweet attraction: Sugarcane pollen-associated volatiles attract gravid *Anopheles arabiensis*. *Malar J.* 2018;17(1):90 10.1186/s12936-018-2245-1 29466989 PMC5822481

[40] Wondwosen B Hill SR Birgersson G: A(maize)ing attraction: gravid Anopheles arabiensis are attracted and oviposit in response to maize pollen odours. *Malar J.* 2017;16(1):39. 10.1186/s12936-016-1656-0 28114992 PMC5259891

[41] Paaijmans KP Wandago MO Githeko AK: Unexpected high losses of *Anopheles gambiae* larvae due to rainfall.Carter D, editor. *PLoS One.* 2007;2(11):e1146. 10.1371/journal.pone.0001146 17987125 PMC2063461

[42] Mutuku F Bayoh M Hightower A: A supervised land cover classification of a western Kenya lowland endemic for human malaria: associations of land cover with larval *Anopheles* habitats. *Int J Health Geogr.* 2009;8(1):19. 10.1186/1476-072X-8-19 19371425 PMC2676261

[43] Minakawa N Sonye G Mogi M: Habitat characteristics of *Anopheles gambiae s.s.* larvae in a Kenyan highland. *Med Vet Entomol.* 2004;18(3):301–5. 10.1111/j.0269-283X.2004.00503.x 15347399

[44] McCann RS Ochomo E Bayoh MN: Reemergence of *Anopheles funestus* as a Vector of *Plasmodium falciparum* in Western Kenya after Long-Term Implementation of Insecticide-Treated Bed Nets. *Am J Trop Med Hyg.* 2014;90(4):597–604. 10.4269/ajtmh.13-0614 24470562 PMC3973499

[45] Mathenge EM Misiani GO Oulo DO: Comparative performance of the Mbita trap, CDC light trap and the human landing catch in the sampling of *Anopheles arabiensis, An. funestus* and culicine species in a rice irrigation in western Kenya. *Malar J.* 2005;4:7. 10.1186/1475-2875-4-7 15667666 PMC548676

[46] Homan T Hiscox A Mweresa CK: The effect of mass mosquito trapping on malaria transmission and disease burden (SolarMal): a stepped-wedge cluster-randomised trial. *Lancet.* 2016;388(10050):1193–201. 10.1016/S0140-6736(16)30445-7 27520594

[47] Schoelitsz B Mwingira V Mboera LEG: Chemical mediation of oviposition by *Anopheles* mosquitoes: a push-pull system driven by volatiles associated with larval stages. *J Chem Ecol.* 2020;46(4):397–409. 10.1007/s10886-020-01175-5 32240482 PMC7205850

[48] Mwingira VS Spitzen J Mboera LEG: The influence of larval stage and density on oviposition site-selection behavior of the Afrotropical malaria mosquito *Anopheles coluzzii* (Diptera: Culicidae).Reisen W, editor. *J Med Entomol.* 2020;57(3):657–66. 10.1093/jme/tjz172 31630193 PMC7197694

[49] Koenraadt CJM Takken W: Cannibalism and predation among larvae of the *Anopheles gambiae* complex. *Med Vet Entomol.* 2003;17(1):61–6. 10.1046/j.1365-2915.2003.00409.x 12680927

[50] Munga S Minakawa N Zhou G: Effects of larval competitors and predators on oviposition site selection of *Anopheles gambiae* sensu stricto. *J Med Entomol.* 2006;43(2):221–4. 10.1603/0022-2585(2006)043[0221:eolcap]2.0.co;2 16619602

[51] Kweka EJ Zhou G Gilbreath TM 3rd: Predation efficiency of *Anopheles gambiae* larvae by aquatic predators in western Kenya highlands. *Parasit Vectors.* 2011;4:128. 10.1186/1756-3305-4-128 21729269 PMC3141748

[52] Mokany A Shine R: Competition between tadpoles and mosquito larvae. *Oecologia.* 2003;135(4):615–20. 10.1007/s00442-003-1215-6 12684864

[53] Fillinger U Sombroek H Majambere S: Identifying the most productive breeding sites for malaria mosquitoes in The Gambia. *Malar J.* 2009;8(1):62. 10.1186/1475-2875-8-62 19361337 PMC2674466

[54] Kweka EJ Munga S Himeidan Y: Assessment of mosquito larval productivity among different land use types for targeted malaria vector control in the western Kenya highlands. *Parasit Vectors.* 2015;8(1):356. 10.1186/s13071-015-0968-1 26142904 PMC4491214

[55] St Laurent B Cooke M Krishnankutty SM: Molecular characterization reveals diverse and unknown malaria vectors in the western Kenyan highlands. *Am J Trop Med Hyg.* 2016;94(2):327–35. 10.4269/ajtmh.15-0562 26787150 PMC4751935

[56] Minakawa N Mutero CM Githure JI: Spatial distribution and habitat characterization of anopheline mosquito larvae in Western Kenya. *Am J Trop Med Hyg.* 1999;61(6):1010–6. 10.4269/ajtmh.1999.61.1010 10674687

[57] Machani MG Ochomo E Amimo F: Resting behaviour of malaria vectors in highland and lowland sites of western Kenya: Implication on malaria vector control measures.Frischknecht F editor. *PLoS One.* 2020;15(2):e0224718. 10.1371/journal.pone.0224718 32097407 PMC7041793

[58] Gimnig JE Ombok M Kamau L: Characteristics of larval anopheline (Diptera: Culicidae) habitats in Western Kenya. *J Med Entomol.* 2001;38(2):282–8. 10.1603/0022-2585-38.2.282 11296836

[59] Takken W Knols BGJ: Olfaction in vector-host interactions. *Ecology and Control of Vector-borne Diseases* The Netherlands: Wageningen Academic Publishers;2010;2:438 10.3920/978-90-8686-698-4

[60] Bokore G: Association between graminoids and the prevalence of immature malaria. Harvard Dataverse, V1.2020 10.7910/DVN/NAT0YY

