# Supplementary material for: A cross-sectional observational study investigating the association between sedges (swamp grasses, Cyperaceae) and the prevalence of immature malaria vectors in aquatic habitats along the shore of Lake Victoria, western Kenya
Source: F1000Res. 2020 Oct 1;9:1032. Originally published 2020 Aug 24. [Version 2] doi: 10.12688/f1000research.25673.2 (PMC7551511; doi:10.12688/f1000research.25673.2)
Supplement: Peer review history

# Peer review: Reviewer response for version 2

**DOI:** 10.5256/f1000research.29842.r72328

**Published:** 2020-10-12

**Authors:** Eric Ochomo

**Affiliations:** 1 Centre for Global Health Research, Kenya Medical Research Institute (KEMRI), Kisumu, Kenya

I think the authors have done a good job of revising the manuscript and it is in a indexable format.

Is the work clearly and accurately presented and does it cite the current literature?

Yes

If applicable, is the statistical analysis and its interpretation appropriate?

Yes

Are all the source data underlying the results available to ensure full reproducibility?

Yes

Is the study design appropriate and is the work technically sound?

Partly

Are the conclusions drawn adequately supported by the results?

Yes

Are sufficient details of methods and analysis provided to allow replication by others?

Yes

Reviewer Expertise:

NA

I confirm that I have read this submission and believe that I have an appropriate level of expertise to confirm that it is of an acceptable scientific standard.

# Peer review: Reviewer response for version 1

**DOI:** 10.5256/f1000research.28336.r70204

**Published:** 2020-09-14

**Authors:** Eric Ochomo

**Affiliations:** 1 Centre for Global Health Research, Kenya Medical Research Institute (KEMRI), Kisumu, Kenya

This is an important article that attempts to associate the presence of sedges and the presence of immature malaria vectors along the shores on Lake Victoria on the Rusianga Island. The article is quite informative and can generate a tool that can be instrumental in the fight against malaria. I agree with most aspects of it but would like to suggest some improvements that can make it even look better. Below are some of the areas I noted that needs adjustment.

1. Exploitation of the oviposition cues can be very important in implementing the attract and kill mosquito control technique. The authors should identify and discuss more  chemical cues that would  potentially attract gravid females to lay eggs in the graminoid other than cedrol present in *Cyperus rotundus* in the other members of Cyperaceae family identified in this article.
2. The method used to estimate the perimeter of individual habitats is subject to errors incase two different people are involved since one person’s step cannot be exactly be the same to another ones. I suggest a verifiable method ought to have been used in evaluating habitat sizes.
3. The author indicates that he performed test for abiotic factors like turbidity, pH and temperature only. I think that the parameters were not the only abiotic factors that would influence the distribution of immature stages of malaria vectors. Abiotic factors like Dissolved oxygen (DO), salinity atmospheric pressure could also influence the abundance and distribution of mosquito larvae in the water and ought to have been evaluated.
4. In establishing the coverage of the various graminoid plants in the larval habitats by visually assigning percentages in a look and see manner, I think this is subject to error too in reporting the coverage of each Cyperaceae member. They are supposed to use a more objective method of estimating the abundance of the sedges.
5. There is a contradiction in reporting the result for species found in the habitats densely covered with graminoid vegetation and those that are found in habitats which are sparsely covered by graminoid vegetation and I am not sure if this was an error.
6. The article could use a thorough review for grammatical errors. 

Is the work clearly and accurately presented and does it cite the current literature?

Yes

If applicable, is the statistical analysis and its interpretation appropriate?

Yes

Are all the source data underlying the results available to ensure full reproducibility?

Yes

Is the study design appropriate and is the work technically sound?

Partly

Are the conclusions drawn adequately supported by the results?

Yes

Are sufficient details of methods and analysis provided to allow replication by others?

Yes

Reviewer Expertise:

Medical Entomology, epidemiology

I confirm that I have read this submission and believe that I have an appropriate level of expertise to confirm that it is of an acceptable scientific standard, however I have significant reservations, as outlined above.

# Response

**Published:** 2020-09-29

**Authors:** Getachew Bokore

**Affiliations:** ICIPE, Kenya

We appreciate the constructive comments of the reviewer. Below we provide a point by point response.

 1.    Exploitation of the oviposition cues can be very important in implementing the attract and kill mosquito control technique. The authors should identify and discuss more chemical cues that would potentially attract gravid females to lay eggs in the graminoid other than cedrol present in Cyperus rotundus in the other members of Cyperaceae family identified in this article.

 We included some discussion on this as suggested. 

 2.    The method used to estimate the perimeter of individual habitats is subject to errors incase two different people are involved since one person’s step cannot be exactly be the same to another ones. I suggest a verifiable method ought to have been used in evaluating habitat sizes.

 We clarified in the methods that the perimeter was estimated always by the same person. The perimeter was a relative estimate rather than a precise measure which we did not consider necessary in context of our study.

 3.    The author indicates that he performed test for abiotic factors like turbidity, pH and temperature only. I think that the parameters were not the only abiotic factors that would influence the distribution of immature stages of malaria vectors. Abiotic factors like Dissolved oxygen (DO), salinity atmospheric pressure could also influence the abundance and distribution of mosquito larvae in the water and ought to have been evaluated.

 We included a justification for the selection of the measures in the method section. All habitats surveyed were very similar in their characteristics, hence no major variation was expected; however, it cannot be excluded and we aimed to interpret the work carefully within its discussed limitations. 

 4.    In establishing the coverage of the various graminoid plants in the larval habitats by visually assigning percentages in a look and see manner, I think this is subject to error too in reporting the coverage of each Cyperaceae member. They are supposed to use a more objective method of estimating the abundance of the sedges.

 Indeed for an ecological mapping of plant cover, there are various methods available for sampling, for example, the quadrant method and similar approaches. We explored and piloted some of these methods prior to the survey, however did not find them very informative or feasible given the nature of habitats. The most common way to measure cover is the visual estimation method. Visual estimation is popular because it is fast, requires no specialized equipment, and can be adapted to plants of various growth forms. Again, we have clarified that the estimation was done by a single person for relative comparability across sites. In the light of our findings, we would not expect that a different method would have led to a different conclusion. 

 5.    There is a contradiction in reporting the result for species found in the habitats densely covered with graminoid vegetation and those that are found in habitats which are sparsely covered by graminoid vegetation and I am not sure if this was an error.

 We were not able to locate the contradiction, possibly this was a misunderstanding?

 6.    The article could use a thorough review for grammatical errors. 

 We have gone through the article and corrected the English for errors.

# Peer review: Reviewer response for version 1

**DOI:** 10.5256/f1000research.28336.r70203

**Published:** 2020-09-08

**Authors:** Sisay Dugassa

**Affiliations:** 1 Vector Biology and Control Research Unit, Aklilu Lemma Institute of Pathobiology, Addis Ababa University, Addis Ababa, Ethiopia

Presentation of the work is very clear with a well designed methodology. Moreover, the analyses and interpretation of the results were properly presented. Importantly, the authors analyzed the association of early instar larvae of *Anopheles* with graminoid plants and reported that “ *Anopheles coustani*, *An. rufipes* and *An. maculipalpis* were found only in aquatic habitats covered with graminoid plants, whereas *An. arabiensis*, *An. ziemanni* and *An. pharoensis* were found in both habitats with and without graminoid plants”. Moreover, they analyzed the correlation between the dominant graminoid plant species and early instar larvae and indicted the correlation is not species dependent. Such results are very important for future work in this area of research. However, there might be less importance of graminoid plants for some *Anopheles* species such as *An. arabiensis*, *An. ziemanni* and *An. pharoensis.* Therefore, authors should clearly indicate the potential importance of other factors than the plant species for the availability and density of the larvae (at least for *An. arabiensis*, *An. ziemanni* and *An. pharoensis*) in their conclusion section.

Is the work clearly and accurately presented and does it cite the current literature?

Yes

If applicable, is the statistical analysis and its interpretation appropriate?

Yes

Are all the source data underlying the results available to ensure full reproducibility?

Yes

Is the study design appropriate and is the work technically sound?

Yes

Are the conclusions drawn adequately supported by the results?

Yes

Are sufficient details of methods and analysis provided to allow replication by others?

Yes

Reviewer Expertise:

Medical Entomologist

I confirm that I have read this submission and believe that I have an appropriate level of expertise to confirm that it is of an acceptable scientific standard.
